# Assessing spectral effectiveness in color fundus photography for deep learning classification of retinopathy of prematurity

**DOI:** 10.1117/1.JBO.29.7.076001

**Published:** 2024-06-18

**Authors:** Behrouz Ebrahimi, David Le, Mansour Abtahi, Albert K. Dadzie, Alfa Rossi, Mojtaba Rahimi, Taeyoon Son, Susan Ostmo, J. Peter Campbell, R. V. Paul Chan, Xincheng Yao

**Affiliations:** a University of Illinois, Chicago, Department of Biomedical Engineering, Chicago, Illinois, United States; b Oregon Health and Science University, Casey Eye Institute, Department of Ophthalmology, Portland, Oregon, United States; c University of Illinois Chicago, Department of Ophthalmology and Visual Sciences, Chicago, Illinois, United States

**Keywords:** retinopathy of prematurity, color channels, fundus imaging, deep learning

## Abstract

**Significance:**

Retinopathy of prematurity (ROP) poses a significant global threat to childhood vision, necessitating effective screening strategies. This study addresses the impact of color channels in fundus imaging on ROP diagnosis, emphasizing the efficacy and safety of utilizing longer wavelengths, such as red or green for enhanced depth information and improved diagnostic capabilities.

**Aim:**

This study aims to assess the spectral effectiveness in color fundus photography for the deep learning classification of ROP.

**Approach:**

A convolutional neural network end-to-end classifier was utilized for deep learning classification of normal, stage 1, stage 2, and stage 3 ROP fundus images. The classification performances with individual-color-channel inputs, i.e., red, green, and blue, and multi-color-channel fusion architectures, including early-fusion, intermediate-fusion, and late-fusion, were quantitatively compared.

**Results:**

For individual-color-channel inputs, similar performance was observed for green channel (88.00% accuracy, 76.00% sensitivity, and 92.00% specificity) and red channel (87.25% accuracy, 74.50% sensitivity, and 91.50% specificity), which is substantially outperforming the blue channel (78.25% accuracy, 56.50% sensitivity, and 85.50% specificity). For multi-color-channel fusion options, the early-fusion and intermediate-fusion architecture showed almost the same performance when compared to the green/red channel input, and they outperformed the late-fusion architecture.

**Conclusions:**

This study reveals that the classification of ROP stages can be effectively achieved using either the green or red image alone. This finding enables the exclusion of blue images, acknowledged for their increased susceptibility to light toxicity.

## 1 Introduction

Retinopathy of prematurity (ROP) occurs in premature infants due to underdeveloped retinal vasculature at birth,^1^ causing abnormal blood vessel growth at the boundary of partially vascularized and avascular retinal areas.^2^^,^^3^ ROP is the leading preventable cause of childhood blindness worldwide.^4^^,^^5^ The severity of ROP is classified into five stages, corresponding to mild to severe stages.^6^ Appropriate detection and accurate ROP diagnosis in premature infants is crucial to prevent irreversible vision loss and associated visual developmental complications such as astigmatism, myopia, glaucoma, cataracts, anisometropia, amblyopia, strabismus, and retinal detachment.^7^^,^^8^

**Fig. 1:**
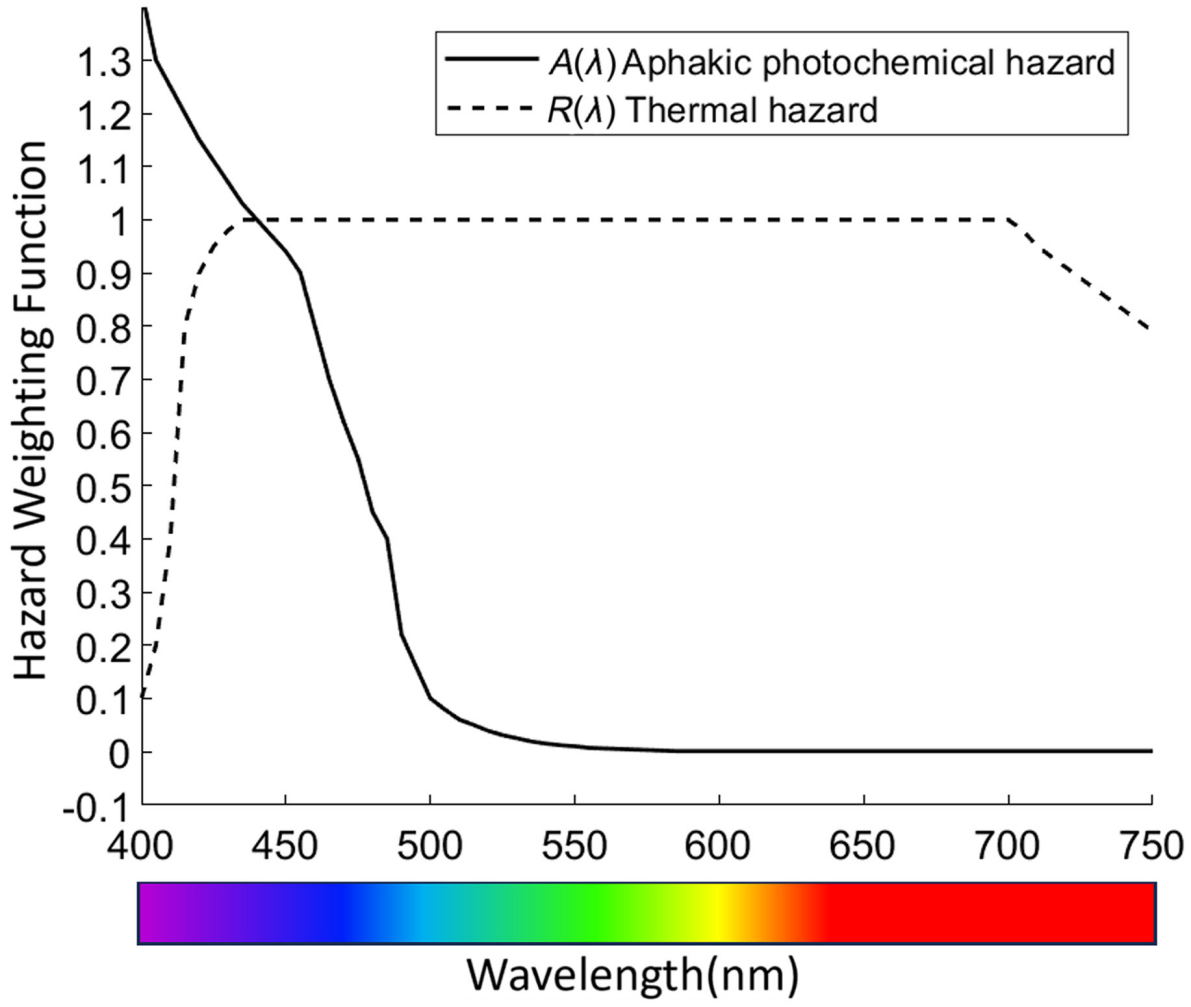
Wavelength-dependent hazard weighting functions based on ISO 15004-2:2007 for aphakic photochemical and thermal hazard factors.

Color fundus photography is widely used for ROP screening and diagnosis.^9^ It involves white light illumination for color fundus imaging. However, long-term exposure of a bright, white light to the examined eye can be stressful for the patient. Moreover, blue spectrum of the white light poses a particular concern for the retina due to its capacity to induce photochemical damage to the cells and structures within the eye.^10^^,^^11^ As illustrated in Fig. 1, when applying a thermal hazard weighting function, the risk of injury appears comparable across blue, green, and red wavelengths. However, when evaluating photochemical hazards, the risk of injury is notably higher for shorter wavelengths, in comparison to longer wavelengths.^12^ In principle, longer wavelength light, such as red and near infrared light, has better illumination efficiency for fundus imaging compared to shorter wavelength light, such as blue color.^13^^–^^15^ Longer wavelengths can penetrate deeper into the ocular tissues due to reduced scattering and absorption coefficients compared to shorter wavelength green and blue light. The wavelength dependent light efficiency can reasonably explain why clinical fundus images are typically red oriented. Multi-spectral fundus photography has revealed that blue and green fundus images are predominantly reflecting retinal layer structure, while red fundus image consists of the information from both retinal and choroidal layers.^16^^,^^17^ In other words, the red image has the potential to convey depth information of the chorioretinal system more effectively than green and blue images. Hence, it is intriguing to explore whether red fundus images can offer adequate information for ROP screening and diagnosis.

Furthermore, the traditional trans-pupillary white light imaging in pediatric fundus assessments faces another challenge, i.e., a limited field of view,^18^ which can lead to extended examination time and difficulty in evaluating the periphery of the retina. The trans pars plana approach has been suggested as a solution, offering an ultra-wide field of view.^19^ The disparate penetration of different white light wavelengths through the sclera, retina, and choroid^15^ has led to the proposal of narrow-bandwidth multispectral imaging as a superior alternative to broad-spectrum white light. With the increasing adoption of widefield multispectral imaging in pediatric fundus evaluations, it is crucial to determine the effectiveness of specific illumination bands in detecting retinal pathologies within this demographic. Given the limited multispectral datasets available for ROP patients, we have segmented existing white light ROP fundus images into their constituent color channels to evaluate whether certain spectra significantly influence the efficacy of classification algorithms. This research is intended to enhance our comprehension of the diagnostic capabilities across various spectral domains, which could inform the selection of optimal wavelengths for future multispectral fundus imaging studies in the pediatric population.

Deep learning, a subset of machine learning, utilizes neural networks for knowledge acquisition from data and performs tasks, such as image classification, segmentation, and detection. It has shown significant advancements in various medical imaging applications.^20^^–^^24^ Previous ROP classification studies mainly focused on direct use of color fundus images,^25^^–^^29^ with a limited exploration of the green channel.^30^^,^^31^ The potential of the red channel for deep learning ROP classification remains unexplored. Considering that the red channel captures information from both the retina and choroid, providing enhanced depth details, we hypothesize that using the red channel alone may offer sufficient information for effective deep learning ROP screening. This study systematically evaluates the impact of individual-color-channel inputs and multi-color-channel fusion options on deep learning ROP classification. In this study, we provide a comprehensive contribution, including a systematic evaluation of the impact of individual color channels on deep learning for ROP classification. Additionally, we assess the efficacy of combining different color channels in fundus imaging by employing fusion strategies within deep learning frameworks.

## 2 Methods

### 2.1 Data Acquisition and Pre-processing

**Fig. 2:**
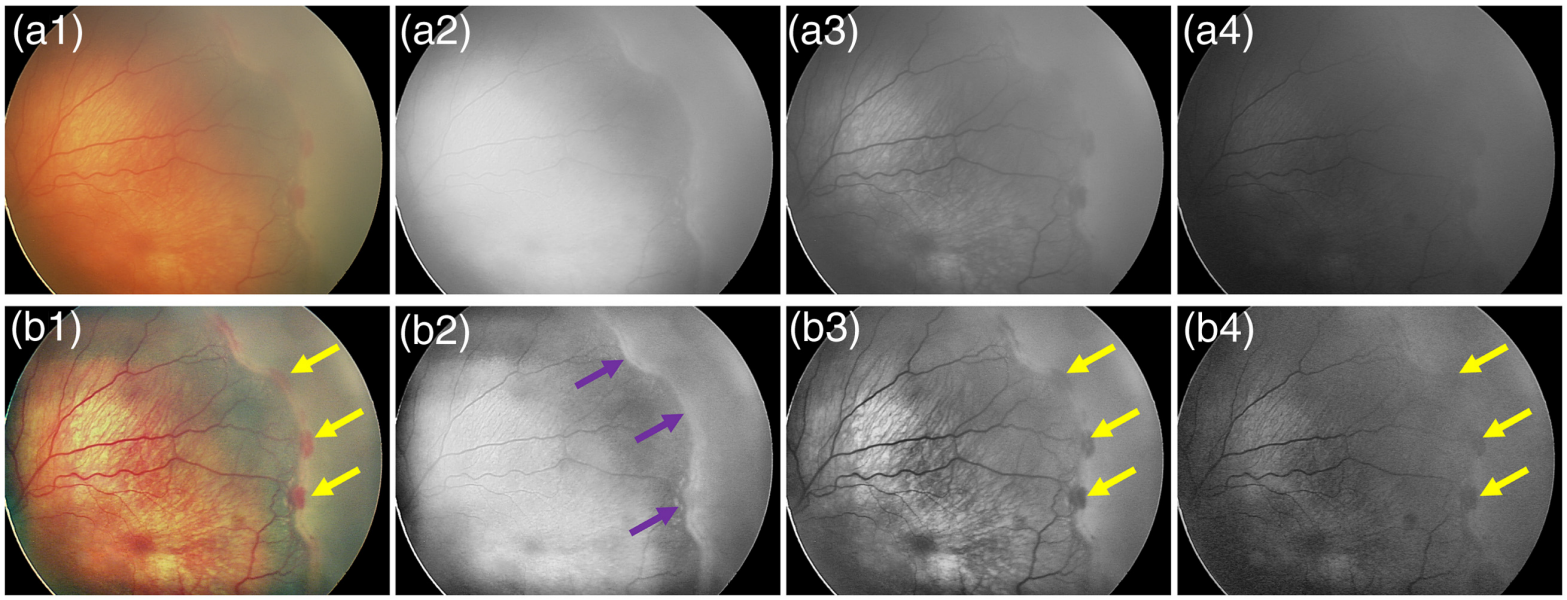
(a) Representative ROP images of color fundus (a1), red (a2), green (a3), and blue (a4) channels of a stage 3 patient. (b) Representative preprocessed ROP images of color fundus (b1), red (b2), green (b3), and blue (b4) of a stage 3 patient.

This study was conducted in accordance with the ethical standards outlined in the Declaration of Helsinki and was approved by the institutional review board of the University of Illinois Chicago. We utilized a dataset comprising 200 color fundus images, distributed among four cohorts (normal, stage 1 ROP, stage 2 ROP, and stage 3 ROP), with 50 images in each category. This dataset consisted of 158 distinct subjects, contributing images from a total of 200 eyes. These images were from the dataset developed by the imaging and informatics for ROP consortium (i-ROP). All images were obtained using the RetCam imaging system, with white and blue LED light sources. The spectral response of the blue, green, and red channels ranges from 400 to 500 nm, 500 to 600 nm, and 600 to 700 nm, respectively.^32^^,^^33^ The dimensions of the original color images were 1600×1200×3 for 37 images and 640×480×3 for 163 images. As illustrated in Fig. 2(a), images of the red channel [Fig. 2(a2)], the green channel [Fig. 2(a3)], and the blue channel [Fig. 2(a4)] were separated from each color fundus image [Fig. 2(a1)] for evaluating the effects of individual-color-channel images on deep learning ROP classification. Moreover, the contrast limited adaptive histogram equalization (CLAHE) technique^34^ was utilized to enhance image contrast. Each image is divided into 8×8 sub-frames, with a contrast enhancement limit of 0.01, a distribution of uniform, and the number of bins set to 256. CLAHE operates by dividing the image into smaller blocks and then applies histogram equalization to each block, limiting the contrast enhancement within a specific block to avoid overamplification of noise. This adaptive approach is particularly effective in enhancing local contrast, which is vital for robust image analysis and classification. Figure 2(b) shows CLAHE-enhanced version of the raw images in Fig. 2(a). The arrows in Fig. 2 demonstrate the fibrovascular ridge region, the critical area observed in ROP.

**Fig. 3:**
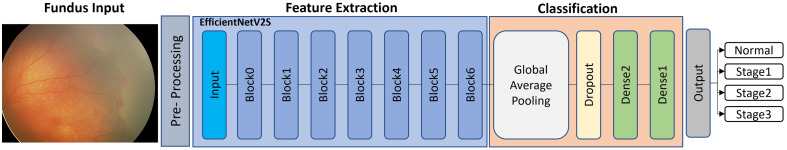
CNN-based end-to-end classifier for ROP classification.

**Fig. 4:**
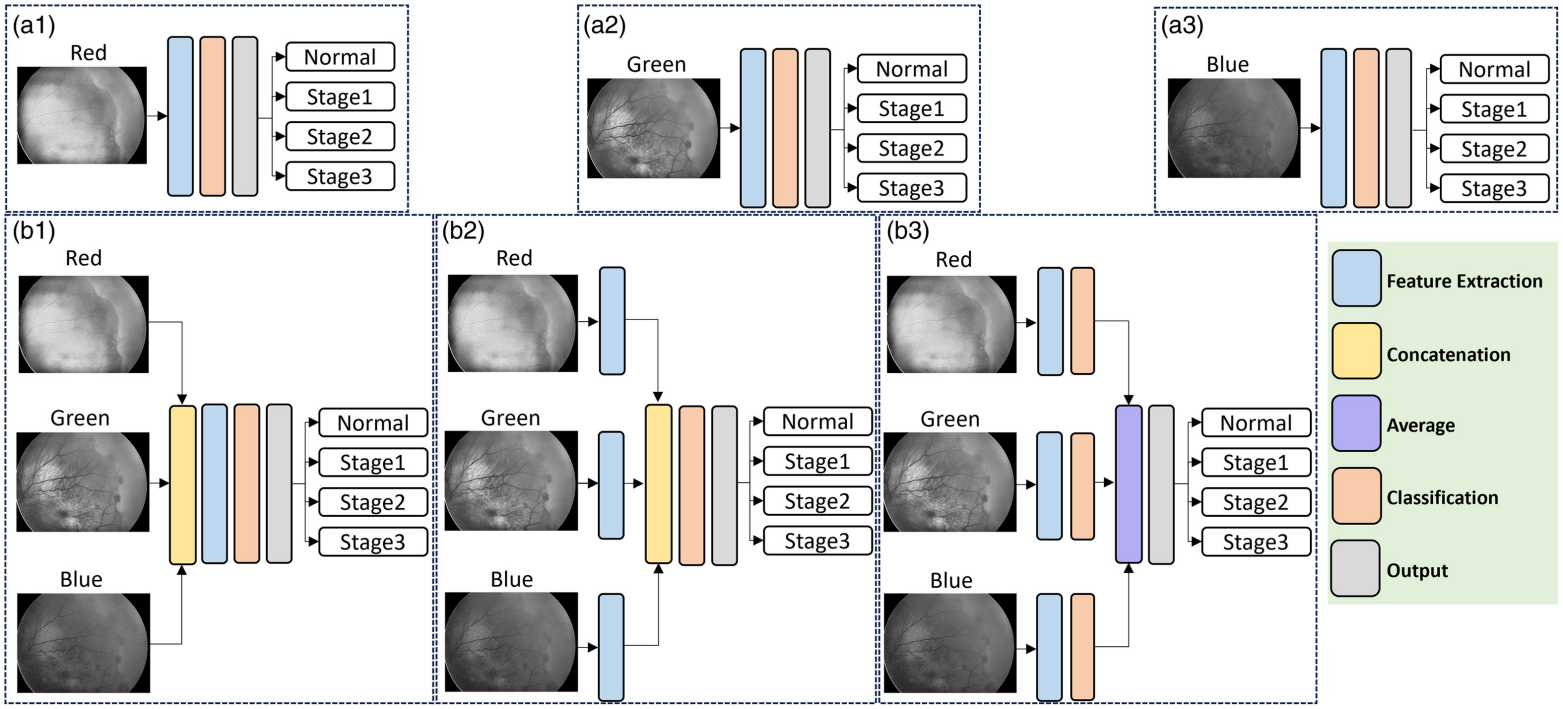
(a) ROP stage classification with red-channel (a1), green-channel (a2), and blue-channel (a3) architectures. (b) ROP stage classification with early-fusion (b1), intermediate-fusion (b2), and late-fusion (b3) architectures.

### 2.2 CNN Architecture and Implementation Procedures

All images were normalized to the dimension of 640×480×3 and subsequent division by 255 to ensure that pixel values were constrained between 0 and 1 prior to being supplied to the model. The base architecture selected for this study was EfficientNetV2S.^35^ As illustrated in Fig. 3, the convolutional neural network (CNN)-based end-to-end classifier for ROP stage classification can be segmented into two parts: features are extracted from the ROP fundus images in the first part, and these features are employed in the second part to classify the images into their respective groups. Two dense layers were utilized, with the first layer comprising 1000 nodes and the second layer consisting of 4 nodes. Transfer learning was employed to address the limited dataset size of available ROP fundus images.^36^ Transfer learning, a training approach, utilizes certain weights from a pretrained CNN to retrain specific layers of the network. After transferring the pre-trained weights, fine-tuning was applied with the available ROP dataset, refining the CNN-based end-to-end classifier further. For all experiments except for the late-fusion, the pretrained weights from the ImageNet dataset were transferred to the EfficientNetV2S base model.^37^ In the late-fusion experiment, the pretrained weights from the individual-color-channel inputs, were utilized to mitigate overfitting and advance generalizability, data augmentation operations, encompassing random rotation, brightness adjustment, horizontal and vertical flipping, zooming, and scaling, were applied. Additionally, a dropout layer with a dropout rate of 10% was employed in the model to prevent overfitting. The training was done for 200 epochs, adopting a learning rate of 0.00001, employing Adam as the optimizer, utilizing categorical cross entropy as the loss function, maintaining a batch size of 32, and incorporating early stopping as a callback function. Early stopping monitors validation accuracy and halts training if it does not improve or worsens for 70 consecutive epochs, reverting to the best observed weights. Given the constrained dataset size, a fivefold cross-validation approach was implemented. Each fold involved training the network with 80% of the images, while the remaining 20 percent were reserved for validation. This approach enabled an evaluation of the model’s performance across a diverse array of images and provided an estimation of the model’s generalizability to novel data.

The model was implemented using Python v3.8 software with the Keras 2.9.0 and TensorFlow 2.9.1 open-source platform backend. Training was performed on a Linux Ubuntu computer with an NVIDIA RTX A6000 graphics processing unit.

### 2.3 Deep Learning ROP Classification with Individual-Color-Channel Images

Figure 4(a) illustrates deep learning ROP classification with individual-color-channel inputs. The individual-color-channel input architectures are defined as red-channel [Fig. 4(a1)], green-channel [Fig. 4(a2)], and blue-channel [Fig. 4(a3)], based on the utilized input channel. This facilitated the determination of the most informative channel for ROP stage classification and facilitated a comparison of the model’s performance across various fusion alternatives.

### 2.4 Deep Learning ROP Classification with Multi-Color-Channel Fusions

#### 2.4.1 Early-fusion

Early-fusion involves concatenating the data from the red, green, and blue channels of ROP fundus images and presenting them to the model as three separate input channels [Fig. 4(b1)]. By combining the data from different channels at the input level, the model can learn to integrate and exploit the complementary information from the different channels to improve the classification performance.

#### 2.4.2 Intermediate-fusion

Intermediate-fusion combines features derived from the red, green, and blue channels for following processing and classification [Fig. 4(b2)]. Each channel ROP fundus image is first processed separately through a feature extraction module. The outputs of the feature extraction modules are then concatenated and fed into the classification module to produce the final prediction.

#### 2.4.3 Late-fusion

Late-fusion combines the red, green, and blue channels after all processing has been completed. As demonstrated in Fig. 4(b3), this involves the extraction of features and subsequent individual classification for each channel, utilizing the fully processed data from each respective input. The outcomes of the classification modules for the three channels are then combined using a global averaging layer. This layer functions by computing the average of the corresponding feature maps across all three channels. The ultimate prediction is determined based on the integrated outputs derived from all three channels.

Initially, the pretrained weights from distinct individual-color-channel inputs [Figs. 4(a1)–4(a3)], were utilized. In other words, each channel was individually trained, and the weights from each model were subsequently used to load onto each branch of the late fusion model. Hence, the initial weights for late fusion were derived from the individual-color-channel inputs rather than being transferred from the pretrained weights of the ImageNet dataset to the EfficientNetV2S base model. This strategy was adopted due to the model’s inability to converge effectively. Given the scale of the model in late-fusion and the extensive number of parameters to be trained, the model could not be adequately trained with a small dataset without risking overfitting.

### 2.5 Evaluation Metrics of Deep Learning Performance

In this study, the assessment of deep learning model performance and the quantification of accuracy and effectiveness relied on receiver operating characteristic (ROC) curve, area under the curve (AUC), accuracy, sensitivity, and specificity. ROC curve plots the true positive rate (sensitivity) against the false positive rate (1-specificity) at varying classification thresholds. AUC signifies the entire area beneath the ROC curve. A model achieving an AUC of 1 signifies a perfect classifier, whereas an AUC of 0.5 indicates no better performance than random chance. Accuracy serves as an indicator of the overall model performance. Sensitivity measures the proportion of true positives correctly identified, while specificity measures the proportion of true negatives correctly identified. These metrics are formally defined as follows:

$$\text{Sensitivity}=\frac{\mathrm{TP}}{\mathrm{TP}+\mathrm{FN}},$$ (1)

$$\text{Specificity}=\frac{\mathrm{TN}}{\mathrm{TN}+\mathrm{FP}},$$ (2)

$$\text{Accuracy}=\frac{\mathrm{TP}+\mathrm{TN}}{\mathrm{TP}+\mathrm{FP}+\mathrm{TN}+\mathrm{FN}},$$ (3)

where TP, TN, FP, and FN represent the number of true positives, true negatives, false positives, and false negatives, respectively.

### 2.6 Class Activation Map

The gradient-weighted class activation mapping (Grad-CAM)^38^ was utilized to identify the crucial ROP fundus regions that were most important for the classification decision. The process involved the input image being passed into the pretrained CNN, enabling the extraction of gradient information flowing into the final convolutional layer. Subsequently, this gradient information was employed to generate a class activation map, providing a detailed representation of the significant regions within the ROP fundus image. This resulting class activation map was overlaid onto the original input image, resulting in the creation of a heatmap visualization. This visualization effectively highlighted and emphasized the regions of the image that held the most substantial influence over the classification decision.

## 3 Results

**Fig. 5:**
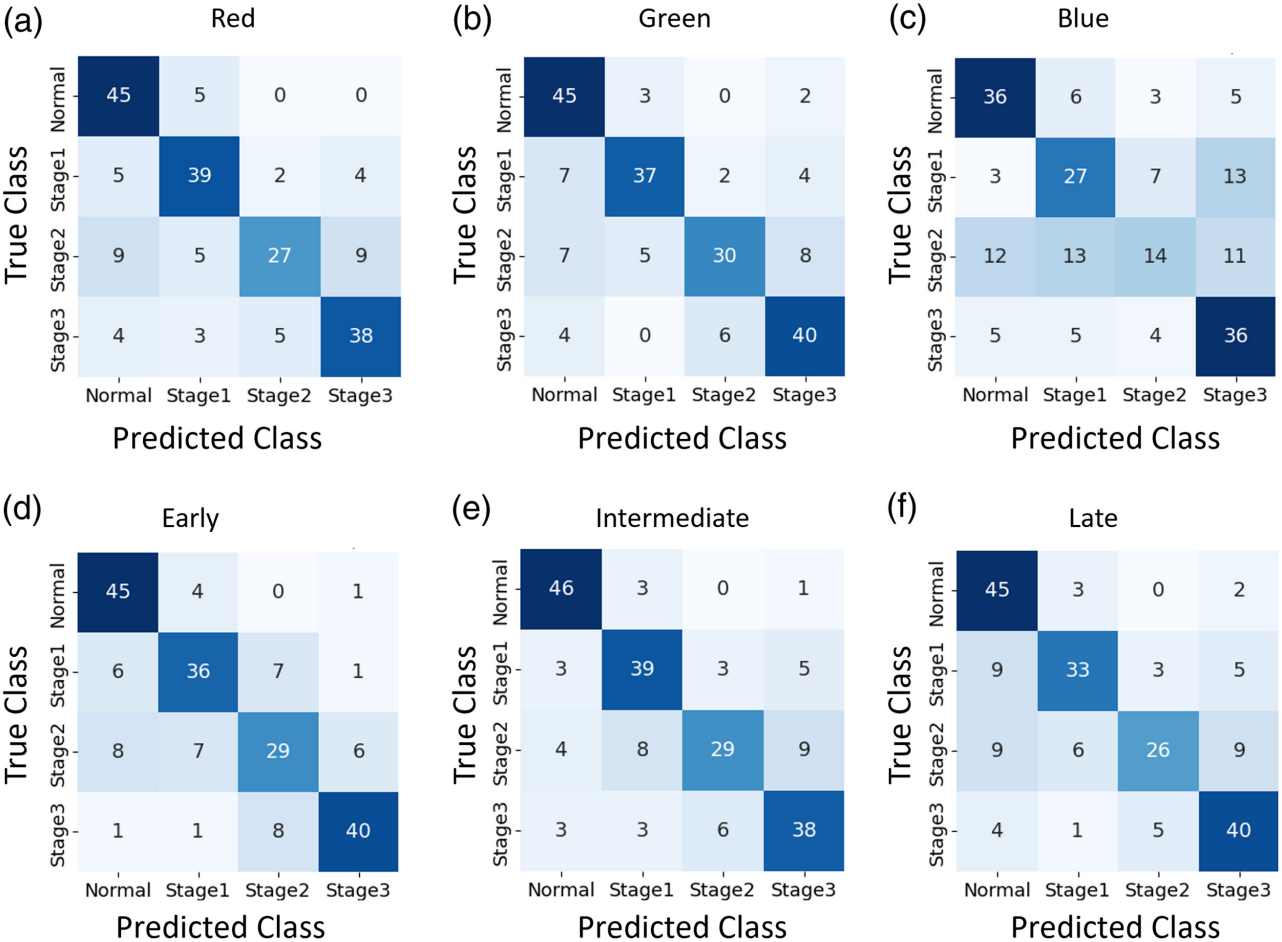
Confusion matrices of red-channel (a), green-channel (b), blue-channel (c), early-fusion (d), intermediate-fusion (e), and late-fusion (f) architectures.

We evaluated the deep learning performance for ROP stage classification using both individual-color-channel inputs and multi-color-channel fusion architectures. The confusion matrices in Fig. 5 illustrate the results for individual-color-channel inputs, i.e., utilizing the red, green, and blue channels [Figs. 5(a)–5(c)], as well as for multi-color-channel fusion options [Figs. 5(d)–5(f)].

Table 1 summarizes the cross-validation performances for both individual-color-channel inputs and multi-color-channel fusion architectures. For individual fundus channel input, Green-channel provided the best performance, with the highest accuracy (88.00%), sensitivity (76.00%), and specificity (92.00%). The red-channel achieved a slightly lower performance compared to the Green-channel with accuracy (87.25%), sensitivity (74.50%), and specificity (91.50%). However, these metrics were notably superior compared to the Blue-channel with accuracy (78.25%), sensitivity (56.50%), and specificity (85.50%). For multi-color-channel fusion options, the early-fusion and the intermediate-fusion architecture showed almost the same performance as green-channel. The late-fusion architecture demonstrated a performance inferior to early-fusion and intermediate-fusion.

**Table 1:** Comparative performance illustration of ROP stage fundus images.

Architectures	Class	Metrics			
		Accuracy mean % (SD)	Sensitivity mean % (SD)	Specificity mean % (SD)	AUC mean (SD)
Red-channel	Normal	88.50 (0.05)	90.00 (0.06)	88.00 (0.54)	0.912 (0.04)
	Stage 1	88.00 (0.04)	78.00 (0.12)	91.33 (0.07)	0.871 (0.04)
	Stage 2	85.00 (0.05)	54.00 (0.24)	95.33 (0.03)	0.786 (0.16)
	Stage 3	87.50 (0.05)	76.00 (0.20)	91.33 (0.08)	0.872 (0.07)
	Average	87.25 (0.05)	74.50 (0.15)	91.50 (0.06)	0.860 (0.08)
Green-channel	Normal	88.50 (0.07)	90.00 (0.09)	88.00 (0.10)	0.931 (0.05)
	Stage 1	89.50 (0.02)	74.00 (0.15)	94.67 (0.06)	0.902 (0.05)
	Stage 2	86.00 (0.05)	60.00 (0.25)	94.67 (0.05)	0.786 (0.14)
	Stage 3	88.00 (0.04)	80.00 (0.11)	90.67 (0.08)	0.896 (0.02)
	Average	88.00 (0.04)	76.00 (0.15)	92.00 (0.07)	0.878 (0.06)
Blue-channel	Normal	83.00 (0.11)	72.00 (0.13)	86.67 (0.11)	0.871 (0.17)
	Stage 1	76.50 (0.06)	54.00 (0.26)	84.00 (0.11)	0.716 (0.12)
	Stage 2	75.00 (0.05)	28.00 (0.18)	90.67 (0.06)	0.566 (0.18)
	Stage 3	78.50 (0.07)	72.00 (0.23)	80.67 (0.17)	0.798 (0.11)
	Average	78.25 (0.07)	56.50 (0.20)	85.50 (0.10)	0.728 (0.15)
Early-fusion	Normal	90.00 (0.04)	90.00 (0.09)	90.00 (0.06)	0.925 (0.05)
	Stage 1	87.00 (0.06)	72.00 (0.23)	92.00 (0.06)	0.911 (0.06)
	Stage 2	82.00 (0.07)	58.00 (0.23)	90.00 (0.06)	0.759 (0.14)
	Stage 3	91.00 (0.04)	80.00 (0.17)	94.67 (0.04)	0.882 (0.07)
	Average	87.50 (0.06)	75.00 (0.18)	91.67 (0.05)	0.869 (0.08)
Intermediate-fusion	Normal	93.00 (0.05)	92.00 (0.07)	93.33 (0.04)	0.950 (0.05)
	Stage 1	87.50 (0.05)	78.00 (0.21)	90.67 (0.08)	0.905 (0.08)
	Stage 2	85.00 (0.07)	58.00 (0.20)	94.00 (0.04)	0.831 (0.16)
	Stage 3	86.50 (0.04)	76.00 (0.14)	90.00 (0.08)	0.891 (0.04)
	Average	88.00 (0.05)	76.00 (0.16)	92.00 (0.06)	0.894 (0.08)
Late-fusion	Normal	86.50 (0.07)	90.00 (0.09)	85.33 (0.09)	0.918 (0.04)
	Stage 1	86.50 (0.03)	66.00 (0.08)	93.33 (0.08)	0.886 (0.05)
	Stage 2	84.00 (0.06)	52.00 (0.20)	94.67 (0.04)	0.725 (0.14)
	Stage 3	87.00 (0.06)	80.00 (0.01)	89.33(0.08)	0.927 (0.04)
	Average	86.00 (0.05)	72.00 (0.12)	90.67 (0.07)	0.864 (0.07)

**Fig. 6:**
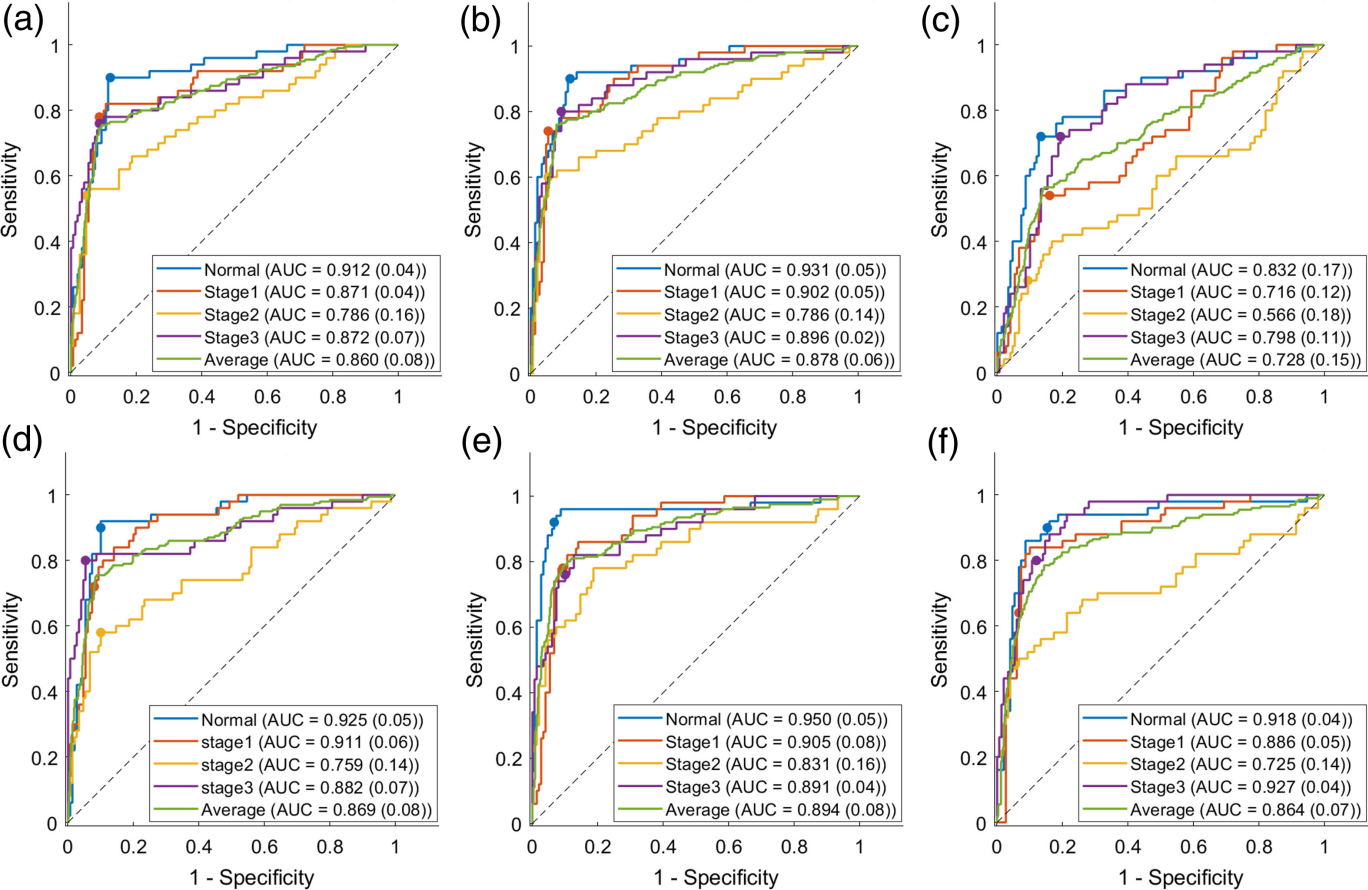
ROC curves of red-channel (a), green-channel (b), blue-channel (c), early-fusion (d), intermediate-fusion (e), and late-fusion (f) architectures with the mean value of the AUCs and their corresponding standard deviations.

Figure 6 illustrates ROC curves of different groups in different architectures. The ROC curves were generated using a one-versus-all approach for each group, aimed at evaluating the classification performance in distinguishing each specific stage from the combined other stages. Each architecture shows the mean value of the AUCs with standard deviations. The average represents the mean value across all four groups. The overall AUC for red-channel, green-channel, blue-channel, early-fusion, intermediate-fusion, and late-fusion are 0.860, 0.878, 0.728, 0.869, 0.894, and 0.864, respectively. Among all architectures, the intermediate-fusion architecture has the highest AUC value. For individual-color-channel inputs, the red-channel and green-channel and blue-channel obtained higher AUC values of the normal group than the stage 1, stage 2, and stage 3 groups. For multi-color-channel fusion options, the early-fusion and the intermediate-fusion obtained higher AUC values of the normal group than the stage 1, stage 2, and stage 3 groups, whereas the late-fusion achieved a higher AUC value of the stage 3 group than the normal, stage 1, and stage 2 groups. In Fig. 6(c), the AUC for stage 2 is 0.566, which shows the Blue-channel is not confident in the prediction of this stage.

To further explore the impact of different channel fusions on ROP stage classification, experiments were conducted using intermediate fusion with only the red and green channels as inputs, excluding the blue channel. The cross-validation performances are summarized in Table 2. A slight improvement in performance was observed compared to using all three channels. This enhancement suggests that the red and green channels provide sufficient and potentially more relevant information for the ROP stage classification.

**Table 2:** Performance of ROP classification for intermediate fusion using red and green channels.

Architectures	Class	Metrics			
		Accuracy mean % (SD)	Sensitivity mean % (SD)	Specificity mean % (SD)	AUC mean (SD)
Intermediate-fusion	Normal	95.50 (0.01)	94.00 (0.04)	97.33 (0.01)	0.965 (0.06)
	Stage 1	87.99 (0.02)	88.00 (0.11)	88.00 (0.05)	0.901 (0.07)
	Stage 2	86.00 (0.06)	64.00 (0.14)	93.00 (0.05)	0.802 (0.12)
	Stage 3	89.49 (0.02)	74.00 (0.04)	94.66 (0.04)	0.915 (0.06)
	Average	89.75 (0.03)	80.00 (0.08)	93.25 (0.04)	0.896 (0.08)

**Fig. 7:**
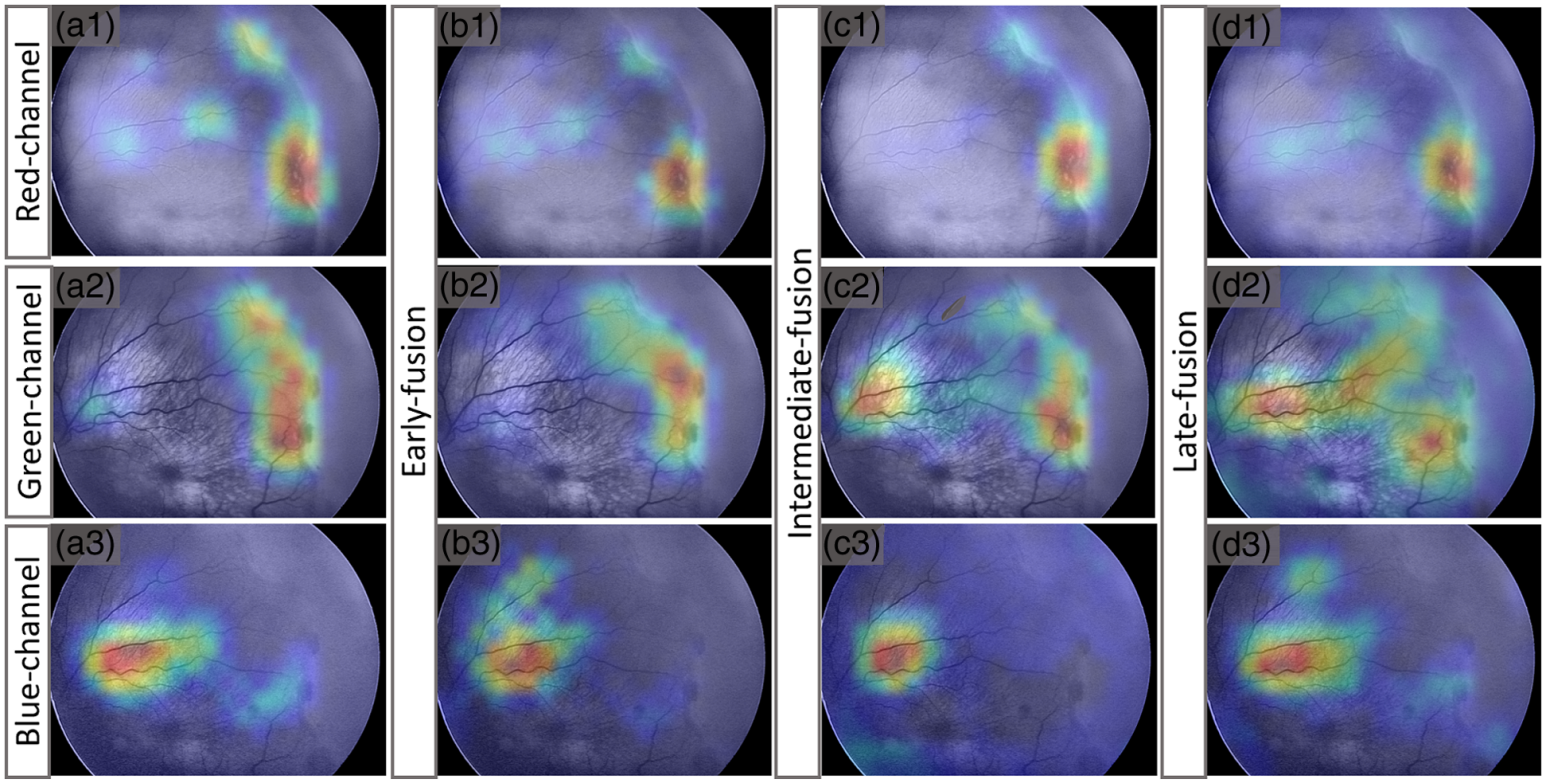
Representative Grad-CAM results for a stage 3 patient [Fig. 2(b)] to highlight the regions useful for deep learning classification in individual-color-channel inputs (a), early-fusion (b), intermediate-fusion (c), and late-fusion (d).

Figure 7 shows representative Grad-CAM maps for a ROP stage 3 patient to highlight the regions useful for deep learning classification for individual-color-channel inputs in red [Fig. 7(a1)], green [Fig. 7(a2)], and blue [Fig. 7(a3)] channels. The Grad-CAM maps confirm that individual color channels can capture different aspects of the ROP abnormalities.

While both the red and green channels effectively identify the fibrovascular ridge region, the classical area observed in ROP, the blue channel predominantly emphasizes the vessels and somewhat neglects this area. When comparing the early-fusion [Fig. 7(b)] and intermediate-fusion [Fig. 7(c)] architectures, it is clear that both fusion architectures maintain the features learned from the individual-color-channel inputs. However, in the late-fusion [Fig. 7(d)] architecture, the model attempts to preserve the features from the individual-color-channel inputs, but it may not prioritize the ridge features as prominently, particularly in the green channel.

## 4 Discussion

We utilized a CNN for end-to-end classification of ROP, covering normal and stages 1 to 3 ROP fundus images. Our evaluation compared classification performance with individual-color-channel inputs and multi-color-channel fusions. Traditional fundus imaging typically employs white light, but it suffers from a reduced field of view. This limitation results in extended examination times and potential difficulties in capturing the retinal edges, risking the oversight of crucial areas that delineate the boundary between vascular and avascular regions critical for detecting ROP stages. To address this, trans pars plana has been proposed to provide the ultimate field of view.^39^ However, due to optical properties, different wavelengths of white light penetrate tissues differently.^15^ In this context, multispectral imaging with narrow bandwidths has been proposed.^17^ Our research enhances our understanding of detection capabilities across diverse spectral ranges and the fusion of different channels, providing insights into the potential benefits of using either single-channel or multispectral imaging for improved diagnostics in ROP. Green-channel demonstrated the highest performance among individual-color-channel inputs, surpassing red-channel marginally and outperforming the Blue-channel. The superiority of the green channel is likely due to its ability to capture critical features in retinal images for ROP stages. Higher absorption rates of blood and hemoglobin in the spectrum around 550 to 580 nm, compared to the spectrum at 420 nm, result in less light reflection from blood-rich areas, making them appear darker on scans and significantly enhancing contrast.^40^ Previous studies^41^^–^^43^ consistently highlight that the green channel of the fundus image offers the highest contrast between retinal vessels and the background. Peng et al.^31^ proposed a deep learning-based ROP staging, incorporating a multi-stream-based parallel feature extractor and a concatenation-based deep feature fuser, utilizing the green channel for per-examination classification of ROP. Similarly, Chen et al.^30^ leveraged the green channel for binary classification of ROP stage while also evaluating the generalizability of deep learning models across various populations and camera systems.

On the other hand, the blue-channel demonstrated a markedly inferior performance compared to the red-channel and green-channel. This can likely be attributed to the limited penetration of short-wavelength blue light into the retina and higher scattering of light, resulting in an inability to effectively capture the deeper retinal layers. Consequently, this limitation led to images with lower contrast between vascular and avascular area, making it challenging to distinguish the main areas, such as the demarcation line or ridge.

In contrast, the red-channel’s performance is comparable to that of the green-channel, early-fusion and intermediate-fusion, underscoring its effectiveness in providing rich spectral information. Red light’s deeper penetration into ocular tissues in a fundus image enables valuable information. Given the association between choroidal and retinal development, it suggests that issues with choroidal development may influence the severity of ROP.^44^^–^^46^ Nonetheless, it is important to acknowledge that the red channel may exhibit reduced contrast, especially when distinguishing between ROP stage 2 and stage 3, where discerning the development of blood vessels in the ridge is crucial. This limitation can make the green channel generally superior. Recognizing the inherent risks associated with shorter wavelengths and the potential discomfort and distress that can be induced by extended exposure to bright white light during eye examinations, using light with longer wavelengths, such as red or green, offers a potentially safer alternative that may also be better tolerated by the infants. It is worth noting that the red channel boasts higher illumination efficiency than the green channel, therefore the power required to do imaging can be reduced drastically compared to green and blue light, making the red channel a safe and practical choice for ROP stage classification. Moreover, transmission efficiency of red spectrum through the sclera is significantly higher than the blue and green spectrum,^47^ making it the optimal choice for trans-scleral illumination.

Our findings highlight the increased difficulty in detecting stage 2 compared to other stages of ROP. Diagnosing stage 2 can be challenging due to the subtle and variable appearance of ridge formation. Clinicians may sometimes misinterpret mild to moderate stage 3 as stage 2, as the extraretinal neovascularization in stage 3 can be difficult to distinguish on 2D images.^48^ Stage 2 often develops popcorn neovascularization, which typically coalesces into the more characteristic appearance of stage 3 and makes the distinction between stage 2 and stage 3 challenging.^49^

Regarding multi-color-channel fusion, early-fusion and intermediate-fusion architectures showed performance comparable to the green-channel, indicating that combining channels did not significantly improve performance beyond that of the green channel alone. This is attributed to the presence of similar biomarkers in both the red and green channels, preventing the intermediate fusion model from leveraging additional distinct information or correlations.

The saliency maps obtained from this analysis reveal a consistent pattern across the red [Fig. 7(a1)] and green [Fig. 7(a2)] channels, indicating a predominant focus on the fibrovascular ridge region and its immediate surroundings, a key sign for ROP stage classification. This suggests that the models can identify key features associated with the presence of ROP stage, highlighting the clinical relevance of these regions in diagnosing these ocular conditions.

However, certain limitations of this study are acknowledged. The relatively small size of the dataset and the lack of external testing cohorts limit the generalizability of the results. Future studies with larger and diverse datasets, including more advanced ROP stages and considering zone or plus disease of ROP, will be crucial for a comprehensive understanding and robust validation of the proposed approaches. Incorporating the near-infrared spectrum also may hold promise for enhanced detection and classification of ROP stages, warranting thorough exploration and validation.

## 5 Conclusion

This study examined the influence of individual-color-channel inputs and multi-color-channel fusions in deep learning for ROP stage classification. The green channel yielded the best performance, slightly surpassing the red channel and significantly outperforming the blue channel. For multi-color-channel fusion options, early-fusion and intermediate-fusion architectures demonstrated nearly matching performance to the green channel input. This comparative analysis suggests that the green or red channel alone can provide sufficient information for ROP stage classification, eliminating the need for blue images. While the study generally concludes that the green or red channel suffices, the red channel may be preferred in specific applications due to its superior illumination efficiency and enhanced transmission through the sclera, offering a safe and practical choice that reduces imaging power requirements and optimizes trans-scleral illumination.

## Data Availability

The code used to generate the results and figures is available in a GitHub repository [https://github.com/geek12b/ROP_EfficientNet_repo]. Data may be obtained from the authors upon reasonable request.
